# GPER-Induced ERK Signaling Decreases Cell Viability of Hepatocellular Carcinoma

**DOI:** 10.3389/fonc.2021.638171

**Published:** 2021-03-09

**Authors:** Yu-an Qiu, Jianping Xiong, Qin Fu, Yun Dong, Manran Liu, Meixi Peng, Wenjian Jin, Lixia Zhou, Xue Xu, Xianming Huang, Airong Fu, Guohui Xu, Gang Tu, Tenghua Yu

**Affiliations:** ^1^Department of Oncology, The First Affiliated Hospital of Nanchang University, Nanchang, China; ^2^Department of Critical Care Medicine, Jiangxi Cancer Hospital, Nanchang University Cancer Hospital, Nanchang, China; ^3^Department of Breast Surgery, Jiangxi Cancer Hospital, Nanchang University Cancer Hospital, Nanchang, China; ^4^Key Laboratory of Medical Diagnostics, Chinese Ministry of Education, Chongqing Medical University, Chongqing, China; ^5^Department of Elderly Oncology, Jiangxi Cancer Hospital, Nanchang University Cancer Hospital, Nanchang, China; ^6^Key Laboratory of Thrombosis and Hemostasis of Ministry of Health, Jiangsu Institute of Hematology, The First Affiliated Hospital of Soochow University, Suzhou, China; ^7^Department of Ultrasonography, Jiangxi Cancer Hospital, Nanchang University Cancer Hospital, Nanchang, China; ^8^Department of Pathology, Jiangxi Cancer Hospital, Nanchang University Cancer Hospital, Nanchang, China; ^9^Department of Hepatobiliary Surgery, Jiangxi Cancer Hospital, Nanchang University Cancer Hospital, Nanchang, China; ^10^Department of Endocrine and Breast Surgery, The First Affiliated Hospital of Chongqing Medical University, Chongqing, China

**Keywords:** G protein-coupled estrogen receptor, cell viability, hepatocellular carcinoma, ERK signaling, therapeutic target

## Abstract

Hepatocellular carcinoma (HCC) is an aggressive malignancy with a poor prognosis. Effective biomarkers and specific therapeutic targets for HCC are therefore urgently needed. G protein-coupled estrogen receptor (GPER) plays a crucial role in numerous cancer types; however, its functions in HCC require further exploration. In the present study, we found a remarkable difference in GPER staining between tumor tissue (100/141, 70.9%) and matched non-tumor tissue (27/30, 90.0%). Compared with the GPER-negative patients, the GPER-positive patients with HCC were closely associated with female sex, negative hepatitis B surface antigen, small tumor size, low serum alpha fetoprotein level, and longer overall survival. Treatment with GPER-specific agonist G1 led to the sustained and transient activation of the EGFR/ERK and EGFR/AKT signaling pathways, respectively, in the HCC cell lines HCCLM3 and SMMC-7721, which express high levels of GPER. Interestingly, G1-induced EGFR/ERK signaling, rather than EGFR/AKT signaling mediated by GPER, was involved in decreasing cell viability by blocking cell cycle progression, thereby promoting apoptosis and inhibiting cell growth. Clinical analysis indicated that simultaneous high expression of GPER and phosphorylated-ERK (p-ERK) predicted improved prognosis for HCC. Finally, the activation of GPER/ERK signaling remarkably suppressed tumor growth in an HCC xenograft model, and this result was consistent with the *in vitro* data. Our findings suggest that specific activation of the GPER/ERK axis may serve as a novel tumor-suppressive mechanism and that this axis could be a therapeutic target for HCC.

## Introduction

Hepatocellular carcinoma (HCC) represents approximately 90% of primary liver cancers and is the third leading cause of cancer-related deaths in China (approximately 422, 100 deaths in 2015) (1, 2). HCC is generally caused by hepatitis B virus infection, hepatitis C virus infection, and alcohol use (3). Surgery, local destructive therapies, and liver transplantation are the current therapeutic strategies for patients with early HCC (4). However, the recurrence rate in treated patients reaches an incidence of >70% at five years (5). Although some treatment processes, including genomic- and immune-based therapies, have been performed in clinical practice, HCC remains one of the deadliest cancers (6). The molecular mechanisms involved in HCC and its potential therapeutic targets must be explored to improve treatment efficacy.

The HCC rate in men is usually 2–4 times higher than that in women, suggesting that sex hormones may play vital roles in HCC pathogenesis (7, 8). Clinical data shows that higher HCC morbidity and mortality occur in male patients, and that estrogen replacement treatment reduces the risk and increases the survival time of female patients with HCC (9, 10).

Estrogen-stimulated actions are mediated by classical nuclear estrogen receptors (ER*α* and ER*β*) (11). GPER, formerly known as G protein-coupled receptor 30, is a non-classical estrogen receptor that rapidly mediates the acute response of effector cells to estrogen and can be specifically activated by G1 (a specific agonist for GPER) (12–14). The activation of GPER can induce multiple downstream effectors, including adenylyl cyclase, epidermal growth factor receptor (EGFR), and phosphoinositide 3-kinase (PI3K), as well as Ca^2+^ mobilization (15–17). Many studies have demonstrated that GPER mediates biological effects in various malignant tumors, including reproductive organs (such as endometrial (18) and ovarian cancers (19)) and other hormonally responsive organs (such as lung (20) and prostate cancers (21)). The biological functions of GPER are inconsistent between different cells and organs. Our previous studies indicated that the activation of GPER enhances the viability of and confers multidrug resistance to breast cancer cells (22–24). Conversely, GPER activation inhibits cell proliferation and increases apoptosis in gastric and colorectal cancers (25, 26). Recent evidence has demonstrated that GPER reprograms the tumor microenvironment, mediates antiviral effects, and suppresses inflammation and fibrosis in HCC (27–29). However, the biological effects and mechanism of action of GPER on HCC cells have not yet been fully explored.

Therefore, the current study aimed to investigate the role of GPER and its potential molecular mechanisms in HCC. We elucidated that the interaction of specific agonist G1-triggered GPER activation and its downstream EGFR/ERK signaling plays a key role in decreasing the tumor viability of HCC. We compared GPER expression in HCC tissue with that in matched non-tumor counterparts and analyzed clinical HCC survival data. We treated two high-GPER-expressing HCC cell lines (*i.e.*, HCCLM3 and SMMC-7721) and a low-GPER-expressing HepG2 cell line with the GPER-specific agonist G1, and measured GPER/EGFR signals and their downstream pathways. We confirmed our findings through immunohistochemical staining of GPER and p-ERK in HCC specimens and *in vivo* xenograft tumors. Our results provide novel insights into GPER-mediated protection against HCC and suggest that targeting the activation of GPER may represent a new therapeutic option for HCC.

## Materials and Methods

### Cell Culture

HepG2, MHCC97-H, and HCCLM3 cells were maintained in Dulbecco’s modified Eagle’s medium (Gibco, MD, USA) supplemented with 10% fetal bovine serum (FBS) (Gibco). SMMC-7721 and HEP3B cells were maintained in RPMI 1640 (Gibco) and Eagle’s minimum essential medium (Gibco), respectively, supplemented with 10% FBS. Before the experiments, all cells were transferred to serum-free medium for 24 h.

### Materials

GPER-specific agonist G1 and GPER-specific antagonist G15 were acquired from Tocris (Ellisville, MO, USA). EGFR inhibitor AG1478 (AG), MEK inhibitor U0126, and PI3K inhibitor wortmannin (WM) were obtained from Millipore (Temecula, CA, USA). The drugs were solubilized in dimethyl sulfoxide (Sigma–Aldrich). The following antibodies were purchased from Bioworld (St. Louis Park, MN, USA) and used for western blotting: ER (1:800), PR (1:500), EGFR (1:1,000), p-ERK (1:1,000), ERK (1:1,000), p-AKT (1:1,000), AKT (1:1,000), p-P38 (1:1,000), P38 (1:1,000), p-JNK (1:1,000), JNK (1:1,000), and p-PKA (1:1,000). GPER (1:250) was obtained from Abcam (Cambridge, MA, USA), and *β*-actin (1:1,000) was purchased from Zhongshan Golden Bridge (Beijing, China).

### Specimens

A total of 141 archival paraffin-embedded HCC specimens and 30 paired normal liver tissue samples were obtained from the Department of Pathology, Jiangxi Cancer Hospital (Nanchang, China). All patients who underwent surgery at the Jiangxi Cancer Hospital between 1999 and 2008 were diagnosed at the same center. The clinical information and overall survival (OS) data of 141 patients with HCC who underwent surgery were collected from the Department of Hepatobiliary Surgery of Jiangxi Cancer Hospital. The study was approved by the Ethics Committee of Jiangxi Cancer Hospital and complied with the Helsinki Declaration.

### Immunoblotting

The cells were treated as follows: (1) cells treated with G1 (1 µM), or (2) before G1 treatment, cells were treated with G15 (1 µM, 30 min), AG (10 µM, 30 min), U0126 (10 µM, 30 min), and WM (10 µM, 30 min). Whole cell extracts were prepared in RIPA buffer with protease inhibitors (Beyotime, Shanghai, China). For the preparation of the membrane and cytosolic fractions, the treated cells were suspended in membrane and cytosolic protein extract buffer (Beyotime) according to the manufacturer’s protocol. Proteins were electrophoresed on a 10% sodium dodecyl sulphate–polyacrylamide gel electrophoresis (SDS–PAGE) gel and transferred onto PVDF membranes. Each primary antibody was incubated with the membranes overnight at 4°C. Membranes were then incubated with the appropriate horseradish peroxidase-conjugated secondary antibody for 1 h at room temperature. The membranes were washed and visualized using an enhanced chemiluminescence system (Amersham Pharmacia Biotech). The intensity of the immunoblot bands was quantified using Quantity One 4.62 software, and results were expressed as fold change relative to the control.

### Cell Transfection

The GPER-specific siRNA (siGPER), non-specific control siRNA, pcDNA (Vector), and pcDNA/GPER (GPER^OE^) were purchased from Genechem (Shanghai, China). HCCLM3 and HepG2 cells were transiently transfected with Lipofectamine™ 2000 reagent according to the manufacturer’s instructions. Target sequences were obtained as previously described (22). The expression level of GPER protein after transfection was measured by western blotting.

### Immunofluorescence

Immunofluorescence assays were performed using a previously described method (30). Cells were grown on sterile coverslips for 24 h, fixed with 4% paraformaldehyde, treated with 0.1% Triton X-100, and blocked with 5% normal goat serum. After blocking, the cells were incubated with the primary antibody (1:200) against GPER at 4°C overnight, washed with phosphate-buffered saline (PBS), and incubated with FITC-labelled goat anti-rabbit secondary antibody (1:200; Zhongshan Golden Bridge) for 30 min at room temperature. Nuclei were stained with DAPI. Immunofluorescence images were collected using the Nikon Eclipse 80i microscope (Tokyo, Japan; 400× magnification).

### Immunohistochemistry

Deparaffinized tissue sections (thickness = 4 μm) were heated at 95°C for 15 min in 10 mM citric acid buffer (pH 6.0) for antigen retrieval and treated with 3% H_2_O_2_ for 10 min to quench the endogenous peroxidase activity. The sections were incubated at 4°C for 16 h with primary antibodies against GPER and p-ERK at a 1:200 dilution. The sections treated with PBS served as the negative control. Sections were treated with horseradish peroxidase-labelled goat anti-rabbit IgG at 37°C for 30 min, followed by diaminobenzidine (Zhongshan Golden Bridge). Slides were counterstained with Mayer’s modified haematoxylin. The immunohistochemistry results were scored independently by two pathologists who were blinded to patient identity using semiquantitative scoring software (Image-Pro Plus 6.0). GPER and p-ERK expression levels were evaluated as previously described (31, 32).

### Flow Cytometry

Flow cytometry (FACSVantage SE; BD, Franklin Lakes, NJ, USA) was used to analyze the cell cycle and apoptosis, as described previously (22, 33). Cells were cultured in FBS-free conditioned medium for 24 h and treated with G1 (1 μM) alone or in combination with G15 (1 μM), AG (10 μM), U0126 (10 μM), and WM (10 μM) pre-treatment for 24 h to detect the DNA content of each phase in the cell cycle. For the cell apoptosis assay, cells were transferred into a conditioned medium without FBS and phenol for 24 h, treated with G1 (1 μM) for 48 h, collected, and stained with Annexin V/PI.

### Cell Viability Assay

A total of 3 × 10^3^ cells were seeded into a 96-well plate and assessed using the CCK-8 protocol (Dojindo Molecular Technologies, Rockville, MD) after treatment. All experiments were performed in duplicate. The absorbance of each well was measured at 450 nm using an enzyme-linked immunosorbent assay microplate reader.

### Reverse Transcription and Real-Time PCR

Total RNA was extracted using TRIzol^®^ (Invitrogen, Carlsbad, CA, USA) and reverse-transcribed using the PrimeScript RT kit (Takara, Dalian, China). Real-time qPCR was performed using SYBR Premix Ex TaqTM II (Takara). The cells were pre-treated to determine the mRNA expression of GPER. Gene expression was calculated using the ΔΔCT method. The primers used for each gene are listed in **Supplementary Table 1**.

### cAMP Measurement

Cells were seeded on 60 mm tissue culture plates at a density of 1 × 10^6^ cells per well for 24 h, switched to a serum-starved medium for 5 h, and treated with G1 as described in the figure legends. Subsequently, the cells were washed twice with PBS, frozen, and thawed three times. The final cAMP levels were measured using the enzyme immunoassay kit (R&D System, Minneapolis, USA) according to the manufacturer’s instructions.

### Xenograft Models

HCCLM3 and SMMC-7721 xenograft cell models were established in athymic nude mice aged 4–6 weeks, which were obtained from the Animal Experimental Center of Chongqing Medical University (Chongqing, China). Experiments were conducted in accordance with the guidelines on animal care and use established by the Chongqing Medical University Experimental Animal Management Committee. The animal study protocols were approved by the ethics committee of Chongqing Medical University.

A total of 5 × 10^6^ cells were implanted into the left armpit. When tumors grew to 150–200 mm^3^ (5–6 weeks), the mice were randomly assigned to experimental groups (*n* = 5 per group). G1, G15, AG, and U0126 were dissolved and diluted in absolute ethanol. The compounds (10 µl) were added to an aqueous vehicle (90 µl, 0.9% NaCl with 0.1% albumin and 0.1% Tween-20). Ethanol (10 µl) was added to the aqueous vehicle (90 µl) and used as the control group. Mice were subcutaneously injected daily with 0.1 ml of G1 (1 μM) alone, or G1 (1 μM) in combination with the GPER-specific antagonist G15 (1 μM), AG (10 μM), or U0126 (10 μM). Body weights were monitored daily. At the end of the 56 day experiment, tumors were removed and measured using a Vernier calliper, and tumor volumes were calculated using the following equation: *Tumor voulume* = 1/2 × *length* × (*width*)2. The expression of specific proteins was analysed by immunoblotting to determine the effects of treatment.

### Prognostic Database of GPER Expression in Patients With HCC

A prognostic database was accessed online (http://kmplot.com/analysis//) and used to predict the prognostic value of *GPER* gene expression in patients with HCC under different parameters (23). Briefly, each percentile of gene expression between the lower and upper quartiles was calculated, and the best-performing threshold was used as the final cut-off in the univariate Cox regression analysis. The clinical data and gene expression data were integrated using the object-relational PostgreSQL database system (http://www.postgresql.org/).

### Statistical Analysis

Statistical analysis was performed using SPSS software, version 21.0. Data are expressed as the mean ± SD. A two-tailed Student’s *t* test was used to compare two groups, whereas one-way ANOVA followed by the Student–Newman–Keuls *post hoc* test was used to compare multiple groups. The association between GPER and p-ERK expression levels in patients with HCC was evaluated using the χ^2^ or Fisher’s exact test. OS analysis was performed for patients who underwent surgery until they died of HCC, and the OS was calculated using the Kaplan–Meier survival method. Differences between survival curves were tested using the log-rank test. P values < 0.05 were considered statistically significant.

## Results

### GPER Is a Potential Prognostic Marker for Patients With HCC

**Figure 1:**
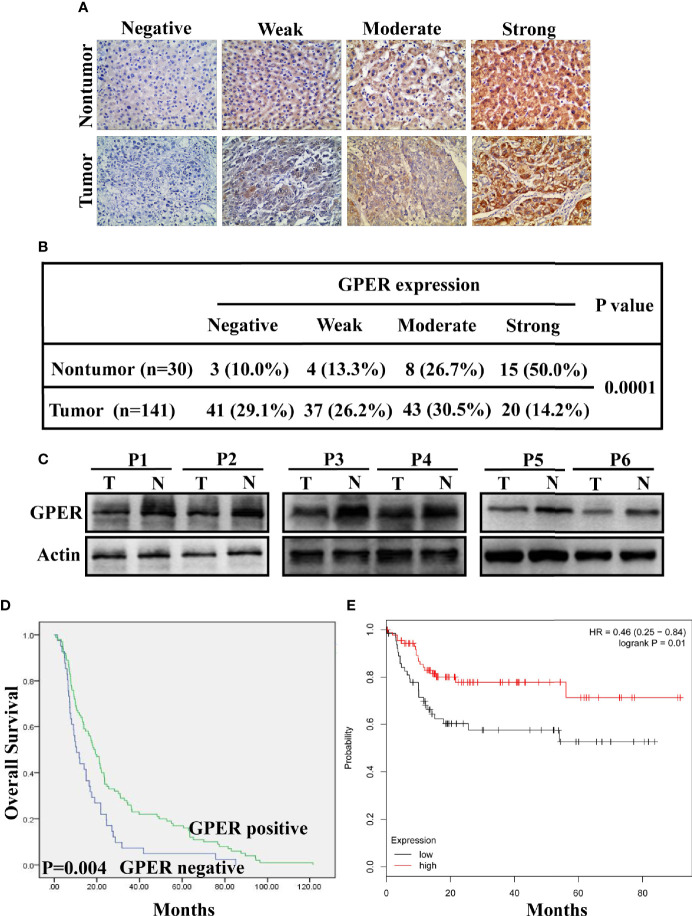
Clinical significance and prognostic value of GPER expression in hepatocellular carcinoma. **(A)** GPER expression (negative, weak, moderate, and strong positive staining) in representative non-tumor tissue (top) and HCC tissue (bottom) was analysed using immunohistochemistry (400× magnification, scale bar = 50 μm). **(B)** IHC analysis revealed significantly different GPER staining between HCC and matched non-tumor tissue (P = 0.0001). **(C)** Western blot of GPER protein in six pairs of frozen HCC tissue (T1–T6) and matched non-cancerous liver tissue (N1–N6). **(D)** High GPER level predicts a good overall survival (OS) in patients with HCC (P = 0.004). **(E)** GPER may be an important protective factor for HCC in Asia (P = 0.01). Data were extracted from a bioinformation database (http://kmplot.com/analysis/).

A total of 141 archival paraffin-embedded HCC specimens and 30 paired non-tumor tissue specimens were eligible for analysis. GPER staining was located in the cytoplasm of both normal liver and tumor tissue (**Figure 1A**). GPER staining was observed in non-malignant tissue with a total positive rate of 90% (27/30), which was significantly higher than that in HCC (70.9%, 100/141; P = 0.0001; **Figure 1B**). Similarly, GPER protein levels in cancerous tissue from six patients with HCC was remarkably decreased compared to in paired non-cancerous tissue (**Figure 1C**).

The correlation between GPER expression and clinicopathological characteristics was retrospectively analyzed to understand the role of GPER in patients with HCC. GPER immunostaining in tumor cells was significantly associated with sex (P = 0.043), negative HBsAg (P = 0.036), small tumor size (P = 0.002), and low serum AFP levels (P = 0.014, **Table 1**). GPER-positive patients showed better OS than GPER-negative patients (P = 0.004, **Figure 1D**). Similar data were also obtained from a bioinformatic database (http://kmplot.com/analysis/) by predicting the prognostic value of GPER in HCC patients from Asia (P = 0.01, **Figure 1E**). These data strongly indicate that GPER is downregulated during the cancer process and could serve as a prognostic marker that also plays a protective role in patients with HCC.

**Table 1:** Correlation between clinicopathological factors and GPER expression in patients with HCC.

Characteristics	Patients (%)	GPER expression		*P* value
		Negative Positive		
Total	141 (100)	41 (29.1%)	141 (70.9%)	
**Sex**				**0.043**
Male	126 (89.4)	40 (31.7%)	86 (68.3%)	
Female	15 (10.6)	1 (6.7%)	14 (93.3%)	
**Age**				0.415
<50	75 (53.2)	24 (32.0%)	51 (68.0%)	
≧50	66 (46.8)	17 (25.8%)	49 (74.2%)	
**HBsAg**				**0.036**
Negative	18 (12.8)	9 (50.0%)	9 (50.0%)	
Positive	123 (87.2)	32 (26.0%)	91 (74.0%)	
**Node Status**				0.430
Negative	131 (92.9)	37 (28.2%)	94 (71.8%)	
Positive	10 (7.1)	4 (40.0%)	6 (60.0%)	
**Tumor size**				**0.002**
T (<5 cm)	80 (56.7)	15 (18.8%)	65 (81.2%)	
T (≧5 cm)	61 (43.3)	26 (42.6%)	35 (57.4%)	
**LDH**				0.085
<225 U/L	71 (50.4)	16 (22.5%)	55 (77.5%)	
≧225 U/L	70 (49.6)	25 (35.7%)	45 (64.3%)	
**AFP**				**0.014**
≦400 ng/ml	81 (57.4)	17 (21.0%)	64 (79.0%)	
>400 ng/ml	60 (42.6)	24 (40.0%)	36 (60.0%)	
**Cancer embolus**				0.751
Negative	124 (87.9)	35 (28.2%)	89 (71.8%)	
Positive	17 (12.1)	6 (35.3%)	11 (64.7%)	
**Hepatocirrhosis**				0.511
Negative	112 (70.4)	34 (30.4%)	78 (69.6%)	
Positive	29 (20.6)	7 (24.1%)	22 (75.9%)	
**Clinical stage**				0.402
I-II	90 (63.8)	24 (26.7%)	66 (73.3%)	
III-IV	51 (36.2)	17 (33.3%)	34 (66.7%)	

P values <0.05 in bold were considered to be statistically significant.

### GPER Expression in Different HCC Cell Lines

**Figure 2:**
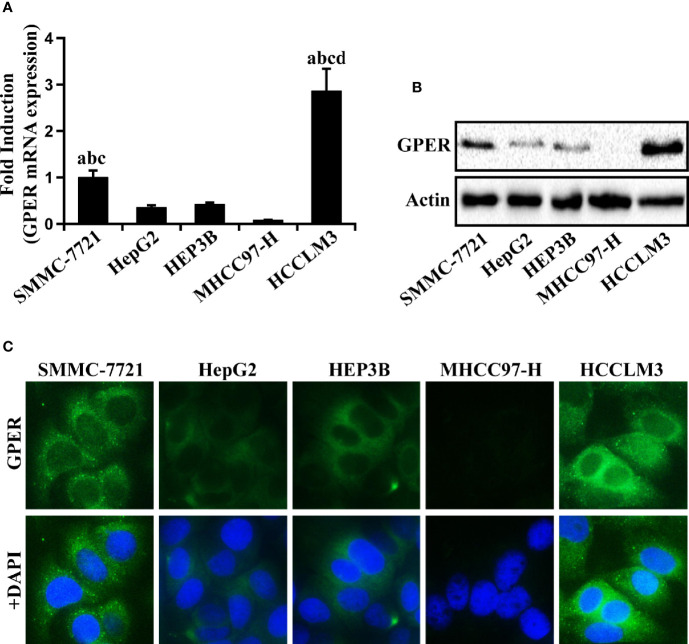
GPER expression in human HCC cells. **(A, B)** Real-time PCR and western blot of GPER in HCC SMMC-7721, HepG2, HEP3B, MHCC97-H, and HCCLM3 cell lines. The relative fold induction of mRNA in each cell line was calculated against the numerical value of GPER expression in SMMC-7721 cells. Data represents the mean ± SD from triplicate independent experiments (P < 0.05; a vs. HepG2; b vs. HEP3B; c vs. MHCC97-H; d vs. SMMC-7721). **(C)** The immunofluorescence localization of GPER (green) viewed in the above five HCC cell lines. The nucleus was stained blue with DAPI (scale bar = 100 μm, 400× magnification). Each experiment was repeated at least three times.

The typical HCC SMMC-7721, HepG2, HEP3B, MHCC97-H, and HCCLM3 cell lines were used to investigate the expression of GPER in multiple HCC cell lines. GPER was expressed at low levels in HepG2, HEP3B, and MHCC97-H cells, but at high levels in HCCLM3 and SMMC-7721 cells (**Figures 2A, B**). The expression pattern of GPER (principally located in the cell cytoplasm) was also confirmed by immunofluorescence (**Figure 2C**) and western blotting (**Supplementary Figure 1A**), which was consistent with the IHC data. We selected high GPER-expressing HCCLM3 and SMMC-7721 cells, and low GPER-expressing HepG2 cells, to further study the role of GPER in HCC *in vitro*.

### Activation of ERK and AKT Is Induced by the GPER-Specific Agonist G1 Through GPER/EGFR Signaling in HCC Cells

**Figure 3:**
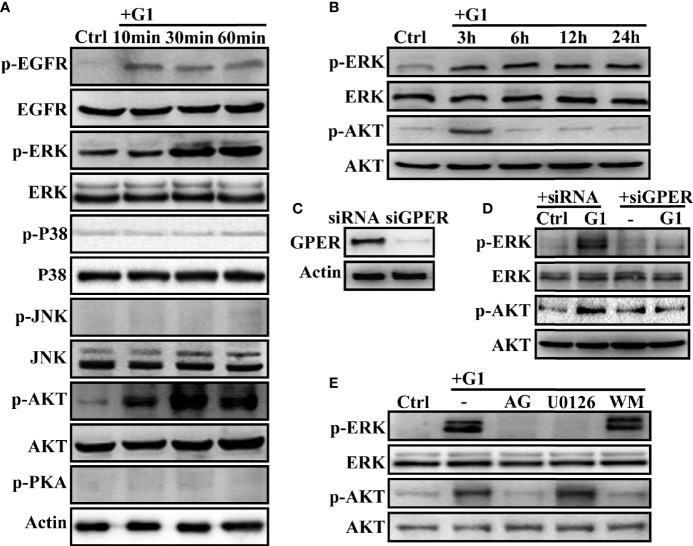
G1 induces the activation of the GPER/EGFR/ERK and GPER/EGFR/AKT signaling pathways in HCC cells **(A)** G1 induced the phosphorylation of EGFR, ERK, and AKT, but not P38, JNK, and PKA in HCCLM3 cells. The HCCLM3 cells were maintained in medium without FBS and phenol for 24 h and treated with 1 μM G1 for 10, 30, and 60 min. **(B)** G1-induced phosphorylation of ERK and AKT was detected for extended periods (3, 6, 12, and 24 h) in HCCLM3 cells. **(C)** GPER expression was knocked down by GPER-specific siRNA transfection of HCCLM3 cells. **(D, E)** G1-induced p-ERK and p-AKT expression levels were blocked by GPER interference or the specific inhibitors AG (1 μM), U0126 (10 μM), and WM (10 μM) in HCCLM3 cells. Each experiment was repeated at least three times.

G1 can trigger GPER/EGFR signaling in malignant tumor cells (22–24, 34, 35). The effects of different doses of G1 on HCC cell growth at different time points were investigated. Since G1 concentrations >1 μM could not further inhibit the growth of HCC cells (**Supplementary Figures 1B, C****)**, 1 μM G1 was chosen for follow-up experiments. We hypothesized that GPER-mediated non-genomic signaling occurs in HCC cells, so the activation of GPER/EGFR signals and their downstream pathways was measured in HCC cells. After a short exposure to G1, significant rapid phosphorylation of EGFR, ERK, and AKT was triggered in HCCLM3 cells (**Figure 3A**). However, G1 had no effect on the activation of P38, JNK, and PKA signaling (**Figure 3A**). The intracellular cAMP levels in HCCLM3 cells were also not affected by G1 (**Supplementary Figure 2A**). Interestingly, the transient activation of AKT signaling was not observed after 3 h of G1 treatment, but a sustained increase in p-ERK expression was detected after 24 h of G1 treatment (**Figure 3B**). These data suggest that G1 enhances ERK and AKT signaling in HCC cells.

Next, the siRNA-specific targeting of GPER (**Figure 3C**), GPER-specific antagonist G15 (1 μM), EGFR inhibitor AG (10 μM), MEK inhibitor U0126 (10 μM), and PI3K inhibitor WM (10 μM) were used to confirm GPER/EGFR/ERK and GPER/EGFR/AKT signaling in HCCLM3 and SMMC-7721 cells. After silencing the *GPER* gene with siRNA or pre-treatment with AG and U0126, the phosphorylation of ERK in HCCLM3 cells was blocked (**Figures 3D, E**). At the same time, p-AKT was significantly blocked by GPER interferents AG and WM (**Figures 3D, E****)**. Similar data were obtained for SMMC-7721 cells (**Supplementary Figures 2B**). However, WM failed to abolish the activation of p-ERK in the two high GPER-expressing cell lines (**Figures 3D, E**, **Supplementary Figure 2B**). Meanwhile, overexpression of GPER in HepG2 cells (**Supplementary Figure 2C**) had similar effects to that in HepG2/GPER^OE^ cells (**Supplementary Figures 2D, E****)**. Taken together, our data demonstrated G1-induced GPER/EGFR/ERK and GPER/EGFR/AKT signaling in HCC cells.

### G1 Decreased the Viability of HCC Cells *via* GPER/EGFR/ERK Signaling

**Figure 4:**
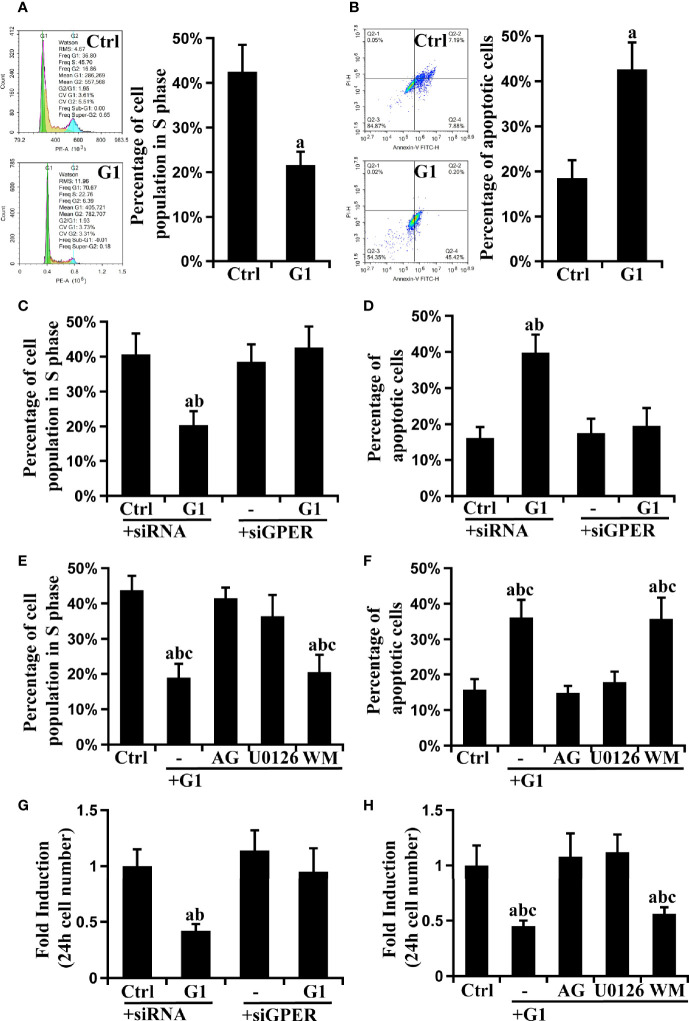
GPER-mediated EGFR/ERK signaling decreases cell viability in HCC cell lines. The GPER-specific agonist G1 blocked progression to the S phase of the cell cycle **(A)** and promoted apoptosis **(B)** in HCCLM3 cells (P < 0.05; a vs. ctrl). Synchronized cells were cultured with G1 and stained with propidium iodide or Annexin V-FITC/PI, and the cell cycle distribution and apoptosis were measured using flow cytometry. **(C, D)** The observed cell cycle and apoptosis blockade by G1 were reversed by GPER interference (P < 0.05; a vs. ctrl + siRNA, b vs. G1 + siGPER). **(E, F)** The observed cell cycle and apoptosis blockade by G1 were attenuated by specific inhibitors targeting EGFR (AG) or ERK (U0126), but not AKT (WM) (P < 0.05; a vs. ctrl, b vs. G1 + AG, c vs. G1 + U0126). The above data represents the mean ± SD from triplicate independent experiments. **(G)** The G1-inhibited cell growth was reversed by GPER interference (P < 0.05; a vs. ctrl + siRNA, b vs. G1 + siGPER). **(H)** The observed inhibition of cell growth by G1 was attenuated by AG and U0126 (P < 0.05; a vs. ctrl, b vs. G1 + AG, c vs. G1 + U0126). The above data represents the mean ± SD from five different experiments.

The effect of GPER signaling on cell cycle and apoptosis was tested using flow cytometry, and cell growth was evaluated using the CCK-8 assay to further explore the role of GPER and its downstream EGFR/ERK and EGFR/AKT signaling pathways in HCC cells. Compared with control HCC cells, G1-treated cells had significantly decreased S phase ratio and increased apoptosis rate (P < 0.05) (**Figures 4A, B** and **Supplementary Figures 3A, B****)**. This phenomenon was reversed after GPER interference or pre-treatment with G15, AG, and U0126 (**Figures 4C–F**, **Supplementary Figures 3C, 3D**). Interestingly, these changes were not observed in WM-treated cells (**Figures 4E, F**, **Supplementary Figures 3C, D****)**. Furthermore, consistent with the results of flow cytometry assays, the inhibitory effect of G1 on cell viability was abolished by the GPER interferents G15, AG, and U0126 (**Figures 4G, H**, **Supplementary Figure 3E**). Similar data were also obtained in HepG2/GPER^OE^ cells (**Supplementary Figures 3F, G****)**. Our results indicate that GPER-induced EGFR/ERK signaling, but not EGFR/AKT signaling, suppressed the viability of HCC cells.

### Co-Expression of GPER and p-ERK Predicts Improved Prognosis for HCC

**Figure 5:**
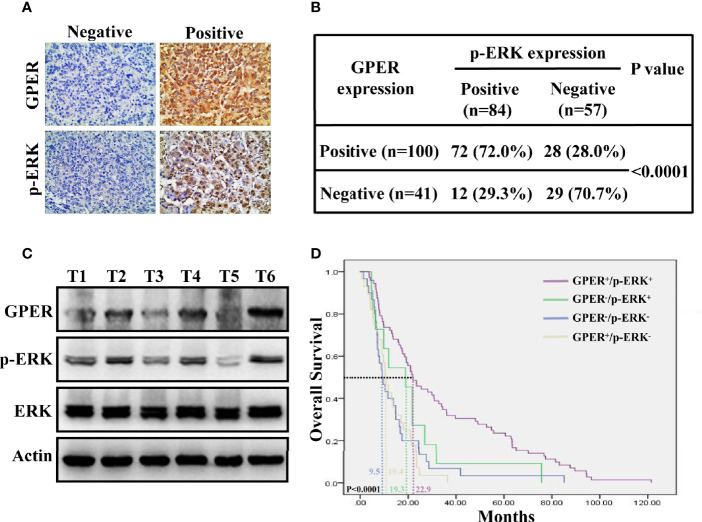
Co-expression of GPER and p-ERK predicts improved prognosis for HCC patients. **(A)** Representative GPER and p-ERK staining in 141 HCC specimens. GPER was detected in the cytoplasm, while p-ERK was mostly expressed in the nucleus (400× magnification, scale bar = 50 μm). **(B)** Significantly different IHC staining of p-ERK in GPER-positive tissue compared with that in GPER-negative tissue (P < 0.0001). **(C)** Western blot of GPER and p-ERK proteins in six frozen HCC tissue samples (T1–T6). **(D)** Kaplan–Meier plots for OS vs. GPER/p-ERK status. Patients with HCC and high GPER/ERK activation are predicted to have better outcomes than those with other subtypes. The median OS times of GPER^-^/p-ERK^-^, GPER^+^/p-ERK^-^, GPER^-^/p-ERK^+^ and GPER^+^/p-ERK^+^ patients were 9.5, 10.4, 19.3 and 22.9 months, respectively (P < 0.0001).

The IHC expression of p-ERK was examined in 141 HCC specimens to further validate the *in vitro* data. GPER was detected in the cytoplasm, whereas p-ERK mostly displayed nuclear staining (**Figure 5A**). GPER and p-ERK were expressed in 70.9% (100/141) and 59.6% (84/141) of tumor tissue, respectively. Furthermore, a significantly different IHC staining of p-ERK was observed in GPER-positive (72/100, 72.0%) and GPER-negative (12/41, 29.3%) HCC tissue (P < 0.0001, **Figure 5B**), indicating that the GPER/ERK pathway was strongly associated with GPER-positive patients. In addition, p-ERK expression was highly consistent with GPER protein levels in cancerous tissue from six patients with HCC (**Figure 5C**). Patients with elevated GPER/ERK signaling activation showed the longest OS time compared to those with other subtypes (P < 0.0001, **Figure 5D**). Thus, our data demonstrated that GPER-mediated ERK signaling might play a protective role in patients with HCC, which is similar to our *in vitro* observations.

### G1-Induced GPER/EGFR/ERK Axis Inhibited the Growth of HCC Xenografts

**Figure 6:**
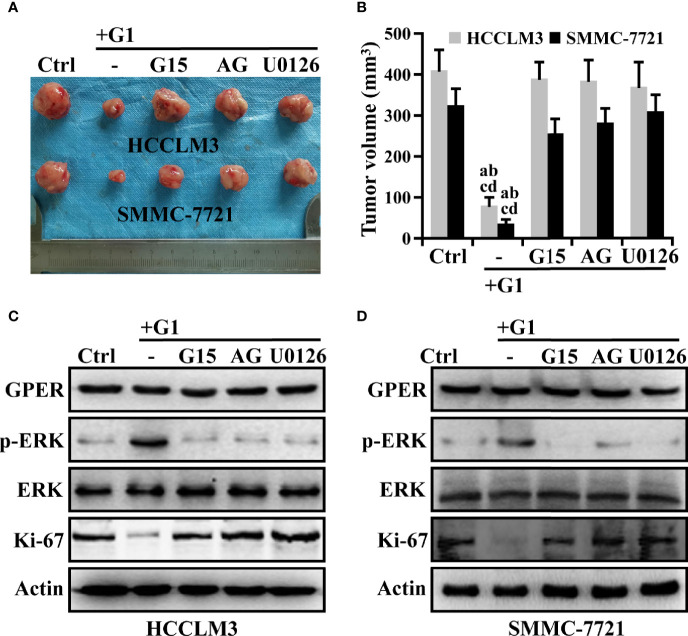
The inhibition of G1-induced GPER/EGFR/ERK signaling abrogates the repression of HCC xenograft growth. **(A, B)** Nude mice bearing HCC xenograft tumors were randomized to receive ethanol alone, G1 (1 μM) alone, or G1 (1 μM) in combination with the GPER-specific antagonist G15 (1 μM), AG (10 μM), or U0126 (10 μM). Images show HCC xenograft tumours in the monotherapy and combination therapy groups. (P < 0.05; a vs. ctrl, b vs. G1 + G15, c vs. G1 + AG, d vs. G1 + U0126). **(C, D)** Western blot detection of the protein expression levels of GPER, p-ERK, and Ki-67 in HCC xenograft tumors. Each experiment was repeated at least three times.

We confirmed that the G1/GPER pathway influenced the viability of HCC cells through EGFR/ERK signaling *in vitro*. HCC xenografts were then used to determine whether this signaling could affect tumorigenesis *in vivo*. Tumors were palpable for approximately 42 days in nude mice. Under treatment, the mean volume of G1 groups decreased by 0.20-fold compared with that of the control group over 56 days (**Figures 6A, B**, P < 0.05). However, the combined pre-treatment with G15, AG, or U0126 remarkably attenuated G1-inhibited growth in HCC xenografts during the intervention (**Figures 6A, B****)**. At the end of drug treatment, the combination groups (G1 + G15, G1 + AG, and G1 + U0126) had at least a fourfold increase in tumor volume compared to the G1 group (**Figures 6A, B****)**. Moreover, these drugs showed no evident toxicity because the body weights of the mice did not change significantly. Western blotting was performed to determine the protein expression levels of GPER, p-ERK, and Ki-67 in drug-treated HCC xenograft tumors. Similar to the *in vitro* data, G1 dramatically improved p-ERK expression, which could be blocked by G15, AG, and U0126, whereas xenograft tumors in all treatment groups possessed almost the same GPER protein levels (**Figures 6C, D****)**. G1 remarkably decreased Ki-67 expression, representing tumor proliferation ability (36), whereas the observed effects could be attributed to the indicated signaling pathway inhibitors (**Figures 6C, D**), and these results corresponded with previous tumor volume studies. These data imply that GPER is a tumor suppressor in HCC xenografts, and that the specific activation of GPER-mediated ERK signaling might present a potential therapeutic avenue for patients with HCC.

## Discussion

Estrogen receptors are thought to regulate HCC tumorigenesis and progression, but the role and mechanism of GPER in the development and progression of HCC have not been thoroughly studied. In the present study, GPER was downregulated in HCC tissue compared with that in matched non-tumor counterparts, and GPER-specific agonist G1-triggered GPER/EGFR/ERK signaling played a crucial role in decreasing the tumor viability of HCC, both *in vitro* and *in vivo*. GPER/ERK signaling is strongly associated with GPER-positive HCC tissue, and patients with simultaneous high expression of GPER and p-ERK showed improved clinical outcomes.

Using a small-scale cohort of 62 HCC samples, Wei et al. showed that GPER staining levels were significantly lower in HCC tissue than in matched non-tumor tissue (29). In the present study, we expanded upon the work by Wei et al. by confirming that GPER is remarkably downregulated in tumor tissue in a larger clinical cohort (141 cases). Moreover, a positive association between GPER expression and several indicators of improved clinical prognosis, such as female sex, negative HBsAg, small tumor size (<5 cm), and low serum level of AFP (≤400 ng/ml), was observed in patients with HCC, and GPER-positive patients exhibited longer OS than GPER-negative patients. These data indicate that GPER may act as a tumor suppressor in HCC. Conversely, Chaturantabut et al. found that human HCC samples (68 cases) have increased GPER expression levels compared with that in non-tumor tissue and that the activation of GPER promotes liver tumor development *via* the PI3K/mammalian target of rapamycin signaling in zebrafish (31). These inconsistent results may be attributed to differences in demographics and clinical sample size between these studies, highlighting the need for large, diverse clinical cohorts in future studies.

The GPER-specific agonist G1, which is wildly employed in the study of numerous cancer types (12, 13, 18–25, 27–31, 34–37), shows extremely high affinity for GPER, but not for classical ERs (14, 38, 39) and 25 other important G-protein coupled receptors (GPCRs) (40, 41), and G1 also had no activity in GPER-knockout mice (42). In the present study, we demonstrate that G1 can block cell cycle progression, promote apoptosis, and inhibit cell growth through GPER/EGFR/ERK signaling in HCCLM3 and SMMC-7721 cell lines. To our knowledge, our work is the first to prove that GPER is a critical factor in these two cell lines. The above data was corroborated by the overexpression of GPER in HepG2 cells. In agreement with our results, a previous study also showed that G1 antagonises the oncogenic actions of leptin in HCC cells by activating GPER/ERK signaling (37). Interestingly, the present study demonstrated that GPER-induced phosphorylation of EGFR, MAPK/ERK, and PI3K/AKT is remarkably upregulated in HCC cells. Despite this, other reported GPER downstream signals, including cAMP/PKA (16, 23), MAPK/JNK (43, 44), and MAPK/P38 (43, 45), were not detected in HCC cells, indicating that GPER may mediate different biological downstream pathways in diverse tumor types. Interestingly, GPER-mediated GPER/AKT signaling is transient, unlike GPER/ERK signaling, and does not contribute to tumor viability in HCC. The definite biological function of GPER-mediated GPER/AKT signaling in HCC should be investigated in detail in the future.

Activation of the ERK signaling pathway is generally associated with enhanced malignant cell survival, metastasis, and clinical drug resistance (46, 47). Targeted inhibition of ERK signaling can suppress hepatocarcinogenesis, and block the invasion and metastasis of HCC (48, 49). However, in the present study, G1 decreased HCC cell viability through the GPER/ERK pathway. Many studies have proposed that the sustained activation of ERK signaling increases cell death *via* cell cycle or apoptosis regulation in a variety of cancer types, including prostate, gastric, colon, and cervical cancers (21, 50–52), which supports our findings. While the overactivation of ERK signaling may be involved in cell death, the appropriate activation of ERK signaling may enhance tumor progression. The balance between the intensity and the duration of pro- or anti-cancer signals transmitted by ERK determines whether tumor cells proliferate or undergo apoptosis.

Our *in vitro* data are strongly supported by *in vivo* results, which show that G1 significantly inhibits tumor growth of HCC xenografts through the GPER/EGFR/ERK axis, and that the specific activation of the GPER/ERK pathway notably enhances tumor reduction. The GPER/ERK pathway is strongly associated with GPER-positive patients with HCC in clinics, and patients with high GPER/ERK activation have better clinical outcomes than those with other subtypes. Our findings further support the idea that the expression of GPER and its downstream signaling should be routinely evaluated in HCC tissue, and the targeted activation of GPER signaling is expected to be effective in improving the prognosis of patients with HCC.

**Figure 7:**
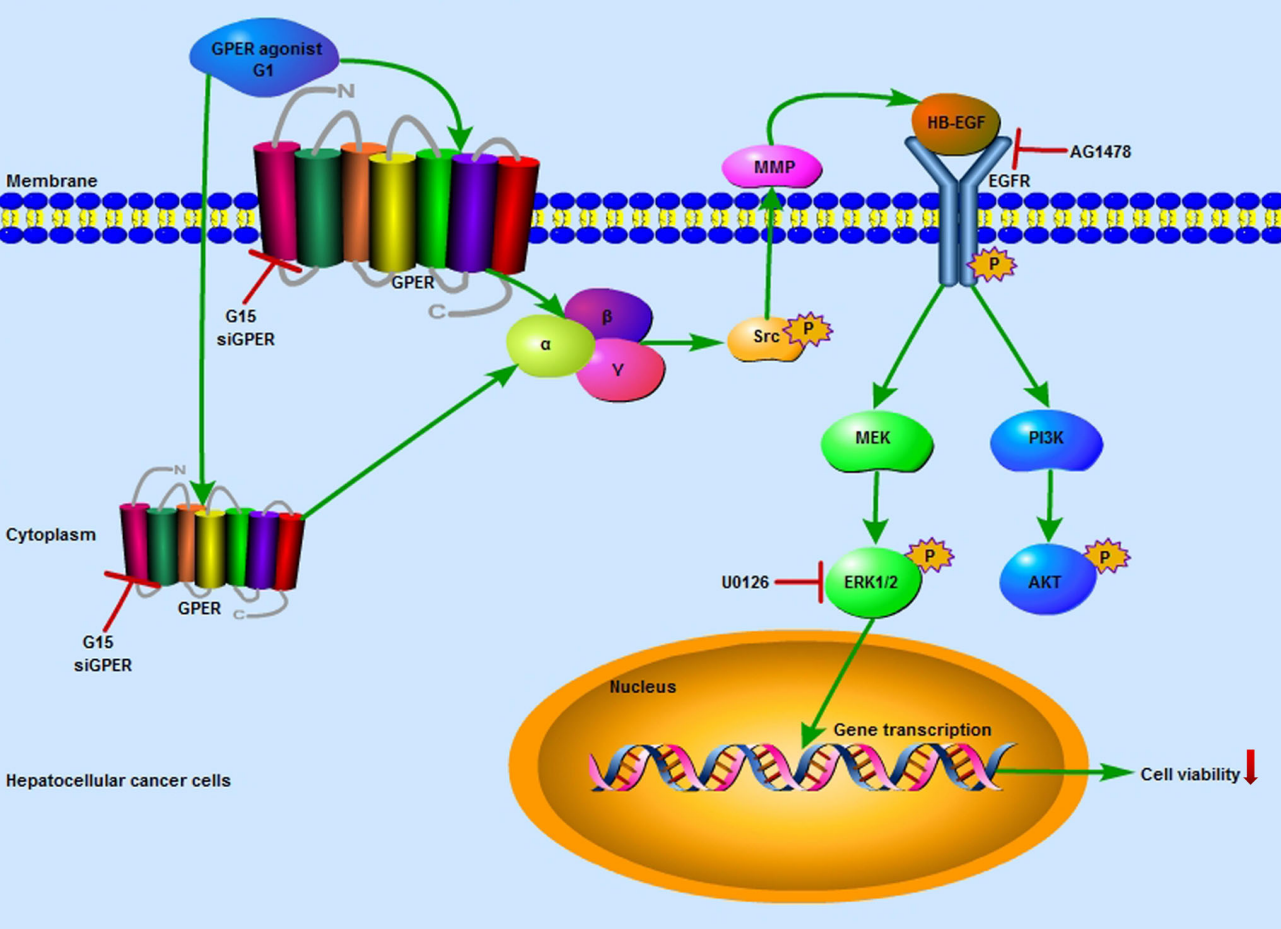
Illustration depicting the specific role of GPER-mediated ERK signaling in decreasing the viability of HCC cells. The GPER-specific agonist G1 promoted crosstalk between GPER and EGFR in HCC cells, and the downstream MAPK/ERK and PI3K/AKT signaling pathways were significantly activated. Activation of the GPER/EGFR/ERK axis blocked cell cycle progression, promoted apoptosis, and inhibited cell growth in HCC cells, finally leading to reduced tumor viability, both *in vitro* and *in vivo*.

In summary, in the present study we provide a novel insight into the role of GPER-mediated tumor suppression in patients with HCC, providing insight into the molecular basis for clinical observations. In brief, the GPER-specific agonist G1 promotes crosstalk between GPER and EGFR in HCC, and the downstream MAPK/ERK and PI3K/AKT signaling pathways are significantly activated. The GPER/EGFR/ERK axis is further responsible for blocking cell cycle progression, promoting apoptosis, and inhibiting cell growth in HCC cells, finally leading to reduced tumor viability both *in vitro* and *in vivo* (**Figure 7**). The activation of GPER/ERK signaling may be a potential treatment for patients with HCC. Further investigations into this signaling cascade, including preclinical and prospective studies, are therefore warranted.

## Data Availability Statement

The original contributions presented in the study are included in the article/**Supplementary Material**. Further inquiries can be directed to the corresponding author.

## Ethics Statement

The studies involving human participants were reviewed and approved by The Ethics Committee of Jiangxi Cancer Hospital. The patients/participants provided their written informed consent to participate in this study. The animal study was reviewed and approved by The Ethics Committee of Chongqing Medical University. Written informed consent was obtained from the individual(s) for the publication of any potentially identifiable images or data included in this article.

## Author Contributions

All authors met the authorship requirements. Y-AQ participated in the design of the study and carried out most of the experiments. JX, QF, YD, ML, and MP participated in immunohistochemistry, RT-PCR, immunoblotting, and immunofluorescence studies. LZ, WJ, and XX participated in siRNA transfection, flow cytometry, CCK8, and *in vivo* assays. XH, AF, GX, and GT analyzed the data and performed the statistical analysis. TY designed the study and drafted the manuscript. All authors contributed to the article and approved the submitted version.

## Funding

This work was supported by the Youths Program of the National Natural Science Foundation of China (81702641) (to TY), the Excellent Youths Program of the Natural Science Foundation of Jiangxi Province (2018ACB21042) (to TY), the Youths Program of the Natural Science Foundation of Jiangxi Province (20171BAB215046) (to TY), and the General Program of the Natural Science Foundation of Jiangxi Province (20192BAB205069) (to Y-AQ).

## Conflict of Interest

The authors declare that the research was conducted in the absence of any commercial or financial relationships that could be construed as a potential conflict of interest.

## Abbreviations

AFP, alpha fetoprotein; AG, AG1478; AKT, protein-serine-threonine kinase; cAMP, cyclic adenosine monophosphate; CCK-8, Cell Counting Kit-8; Ctrl, control; DAB, daiminobenezidine; DAPI, 4,6-diamino-2-phenyl indole; DMEM, Dulbecco’s modified Eagle’s medium; EGFR, epidermal growth factor receptor; ERK, extracellular signal-regulated kinase; ERα, estrogen receptor alpha; ERβ, estrogen receptor beta; FBS, fetal bovine serum; FITC, fluorescein isothiocyanate; GAPDH, glyceraldehyde-3-phosphate dehydrogenase; GPER, G-protein-coupled estrogen receptor; GPR30, G protein-coupled receptor 30; HBsAg, hepatitis B surface antigen; HCC, hepatocellular carcinoma; JNK, c-Jun N-terminal kinases; MAPK, mitogen-activated protein kinases; MEK, mitogen-activated protein kinase/extracellular signal-regulated kinase; mRNA, messenger RNA; OS, overall survival; P38, p38 mitogen-activated protein kinases; PBS, phosphate-buffered saline; PI, propidium iodide; PI3K, phosphatidylinositol 3-kinases; PKA, protein kinase A; RT-PCR, real-time PCR; SDS–PAGE, sodium dodecyl sulphate–polyacrylamide gel electrophoresis; siRNA, small interfering RNA; WM, Wortmannin.
