# Supplementary material for: GPER-Induced ERK Signaling Decreases Cell Viability of Hepatocellular Carcinoma
Source: Front Oncol. 2021 Mar 9;11:638171. doi: 10.3389/fonc.2021.638171 (PMC7985169; doi:10.3389/fonc.2021.638171)
Supplement: Supplementary file 1 [file DataSheet_1.docx]

**Supplementary Materials for**

**GPER-induced ERK signaling decreases the cell viability of hepatocellular carcinoma**

*Yu-an Qiu^1,2^, Jianping Xiong^1^, Qin Fu^3^, Yun Dong^3^, Manran Liu^4^, Meixi Peng^4^, Wenjian Jin^5^, Lixia Zhou^6^, Xue Xu^7^, Xianming Huang^8^, Airong Fu^8^, Guohui Xu^9^, Gang Tu^10^, Tenghua Yu^3^**

^1^Department of Oncology, The First Affiliated Hospital of Nanchang University, Nanchang 330006, China; ^2^Department of Critical Care Medicine, Jiangxi Cancer Hospital, Nanchang University Cancer Hospital, Nanchang 330029, China; ^3^Department of Breast Surgery, Jiangxi Cancer Hospital, Nanchang University Cancer Hospital, Nanchang 330029, China; ^4^Key Laboratory of Medical Diagnostics, Chinese Ministry of Education, Chongqing Medical University, Chongqing 400016, China; ^5^Department of Elderly Oncology, Jiangxi Cancer Hospital, Nanchang University Cancer Hospital, Nanchang 330029, China; ^6^Key Laboratory of Thrombosis and Hemostasis of Ministry of Health, Jiangsu Institute of Hematology, The First Affiliated Hospital of Soochow University, Suzhou 215006, China; ^7^Department of Ultrasonography, Jiangxi Cancer Hospital, Nanchang University Cancer Hospital, Nanchang 330029, China; ^8^Department of Pathology, Jiangxi Cancer Hospital, Nanchang University Cancer Hospital, Nanchang 330029, China; ^9^Department of Hepatobiliary Surgery, Jiangxi Cancer Hospital, Nanchang University Cancer Hospital, Nanchang 330029, China; ^10^Department of Endocrine and Breast Surgery, The First Affiliated Hospital of Chongqing Medical University, Chongqing 400016, China.

**Figure Captions**

**Supplementary Figure 1. Effects of diverse G1 concentrations on the HCC cell growth at different time points.** (A) The GPER protein in the membrane and cytosolic fractions of five HCC SMMC-7721, HepG2, HEP3B, MHCC97-H, and HCCLM3 cell lines were detected by western blot. When the concentration of G1 reached 1 μM, the increase in G1 could not further inhibit the growth of the HCC HCCLM3 (B) and the SMMC-7721 (C) cells (P < 0.05; a vs. Ctrl, b vs. 10 nM, c vs. 100 nM). The above data represented mean ± SD from five independent experiments.

**Supplementary Figure 2. GPER/EGFR/ERK and the GPER/EGFR/AKT signaling, but not the GPER/cAMP signaling, are induced by G1 in HCC cells.** (A) The intracellular cAMP level of HCCLM3 cells was not affected by the G1. (B) The expression levels of the p-ERK and the p-AKT triggered by the G1 in SMMC-7721 cells were inhibited by the specific inhibitors G15, AG, U0126, and WM. (C) GPER was over-expressed by the pcDNA/GPER (GPER^OE^) transfection of HepG2 cells. (D) G1 significantly improved p-ERK and p-AKT expressions only after over-expression of GPER in HepG2 cells. (E) G1-induced p-ERK and p-AKT expressions were blocked through the specific inhibitors G15, AG, U0126, and WM in HepG2/GPER^OE^ cells. Each experiment was repeated at least three times.

**Supplementary Figure 3. G1 decreases the cell viability via the GPER/EGFR/ERK signaling pathway in HCC cells.** The GPER-specific agonist G1 blocked the (A) S phase of the cell cycle progression and promoted the (B) cell apoptosis of SMMC-7721 cells. (C–E) The G1-blocked cell cycle progression, G1-promoted cell apoptosis, and G1-inhibited cell growth were reversed by the specific inhibitor targeting the GPER (G15), EGFR (AG), or ERK (U0126) but not the AKT (WM) in SMMC-7721 cells (P < 0.05; a vs. Ctrl, b vs. G1 + G15, c vs. G1 + AG, d vs. G1 + U0126). (F) G1 significantly enhanced cell apoptosis only after over-expression of GPER in HepG2 cells (P < 0.05; a vs. G1 +Vector, c vs. GPER^OE^). (G) The G1-inhibited cell growth were reversed by the specific inhibitors G15, AG, U0126, and WM in HepG2/GPER^OE^ cells. (P < 0.05; a vs. Ctrl, b vs. G1 + G15, c vs. G1 + AG, d vs. G1 + U0126). The above data represented mean ± SD from at least three independent experiments.

**Supplementary Table 1. Quantitative real-time PCR primers of mRNA.**
