# Supplementary figures and images for: GPER-Induced ERK Signaling Decreases Cell Viability of Hepatocellular Carcinoma
Source: Front Oncol. 2021 Mar 9;11:638171. doi: 10.3389/fonc.2021.638171 (PMC7985169; doi:10.3389/fonc.2021.638171)

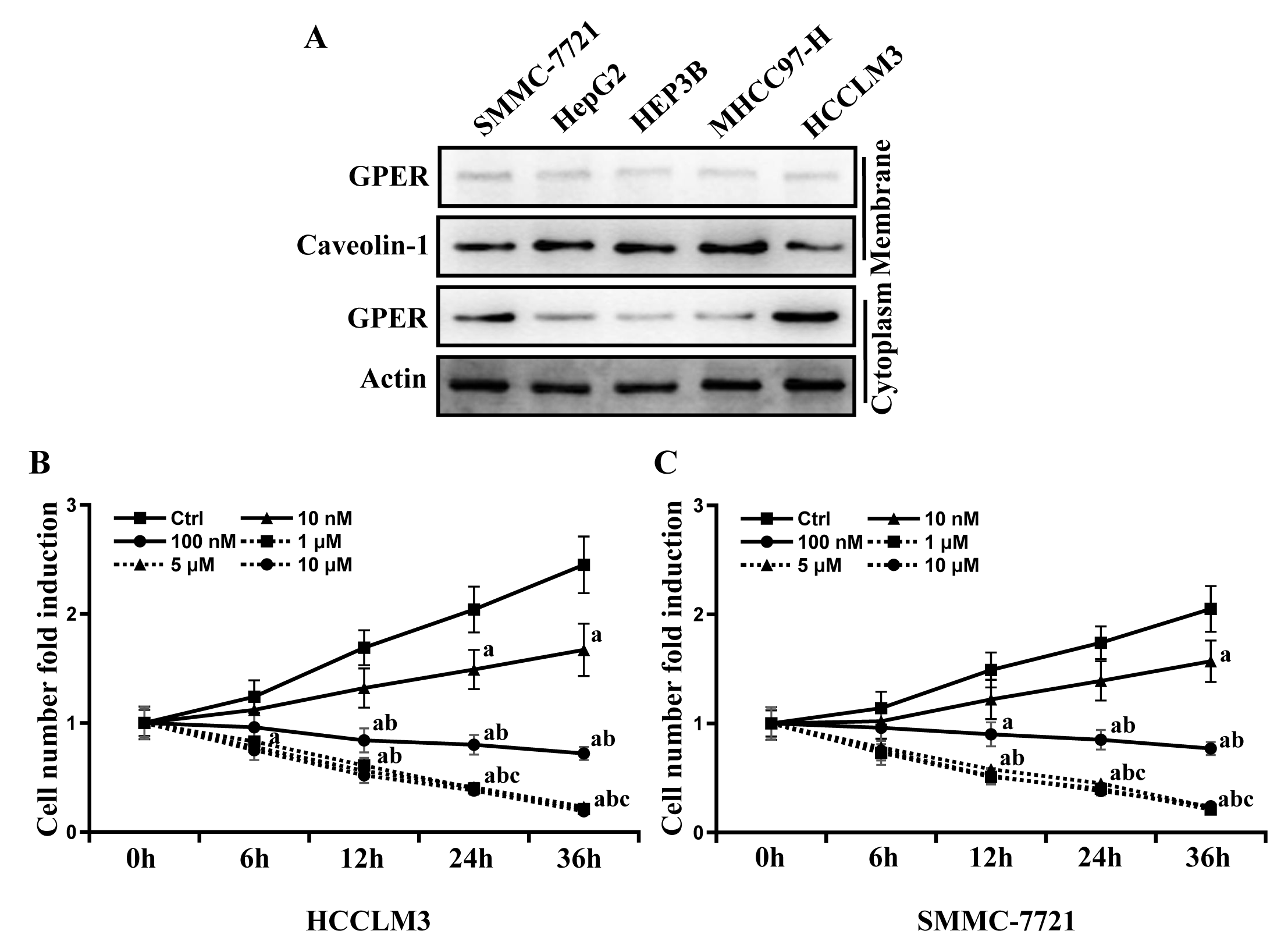

Supplement: Supplementary file 2 [file Image_1.tif]

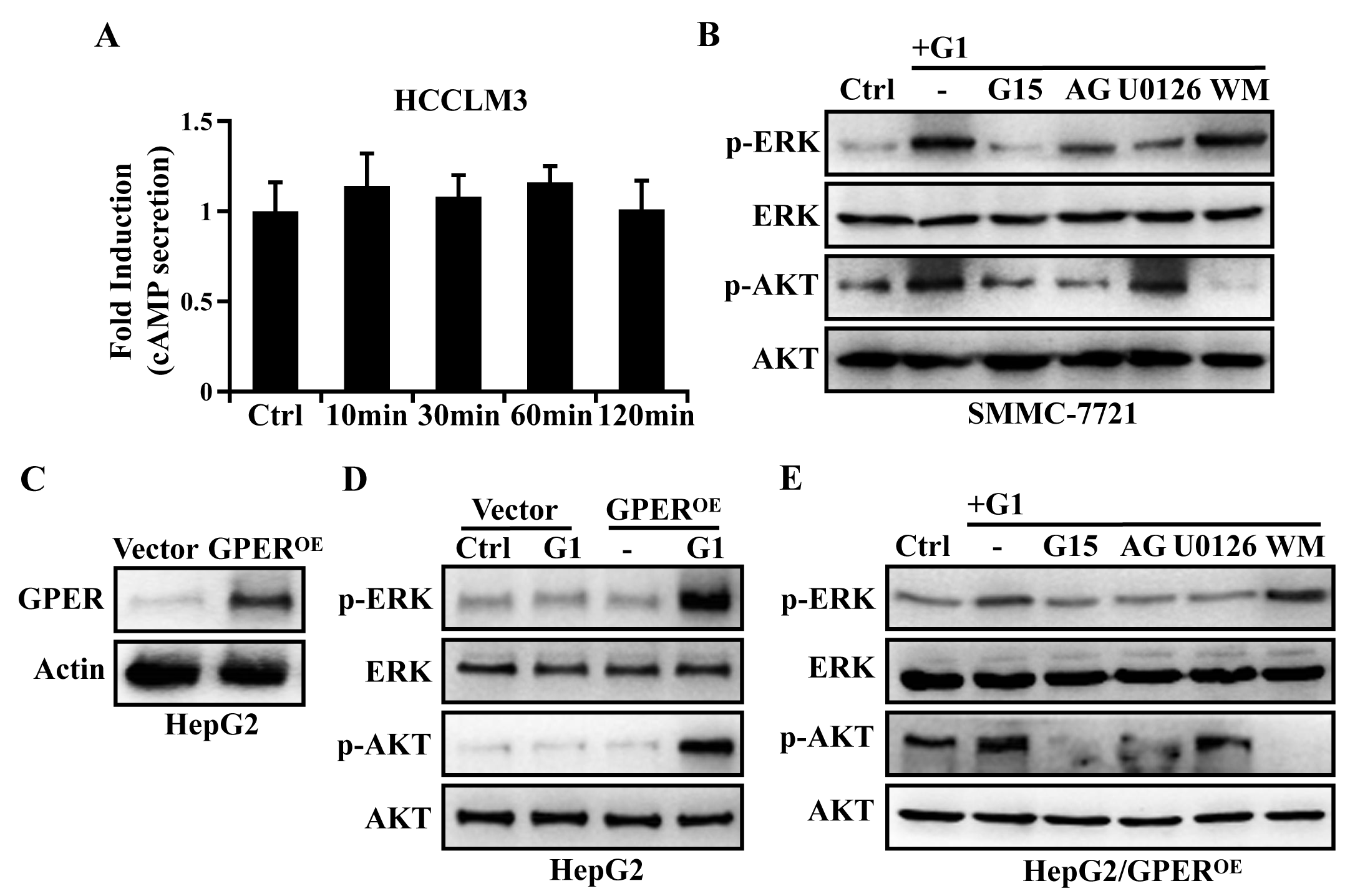

Supplement: Supplementary file 3 [file Image_2.tif]

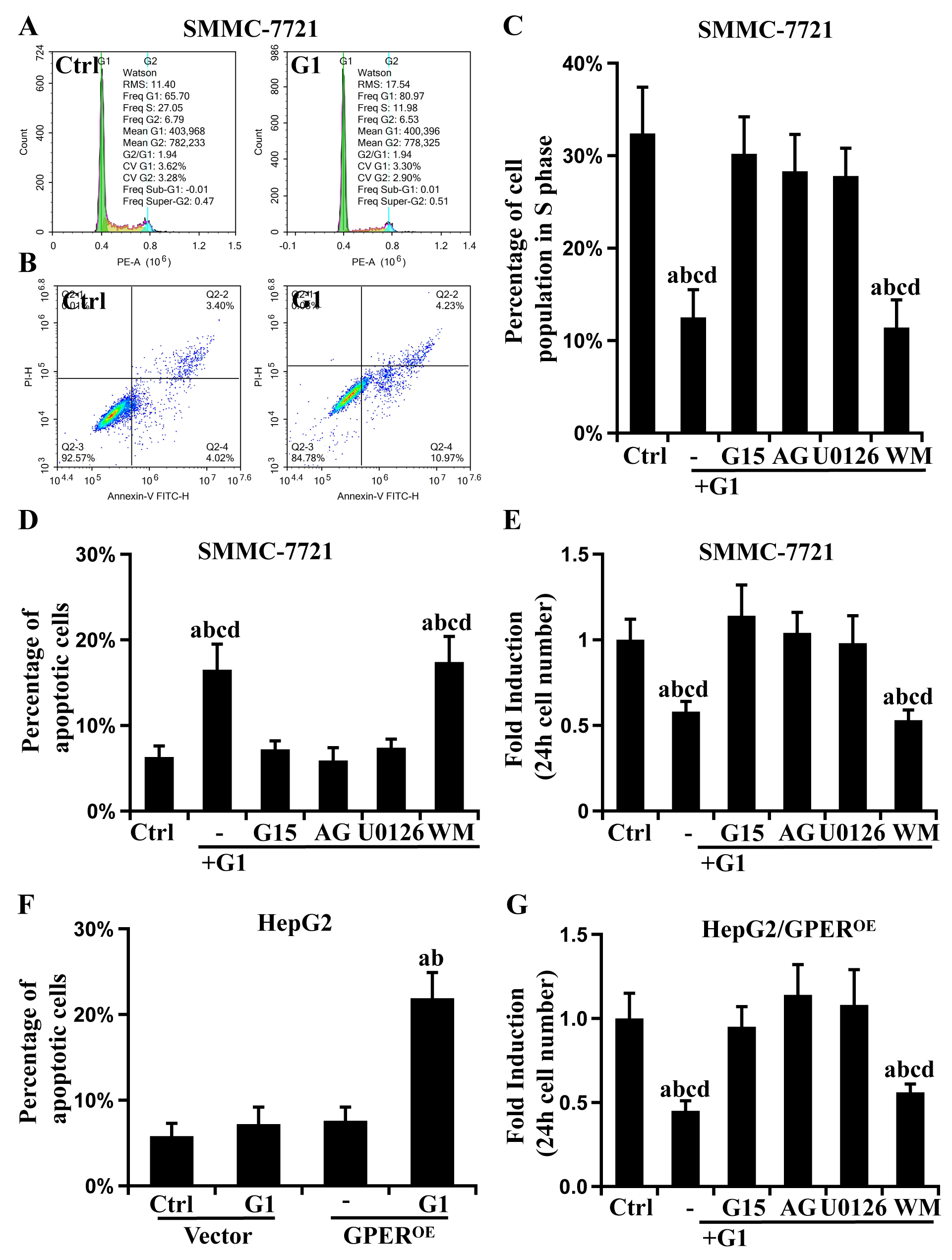

Supplement: Supplementary file 4 [file Image_3.tif]

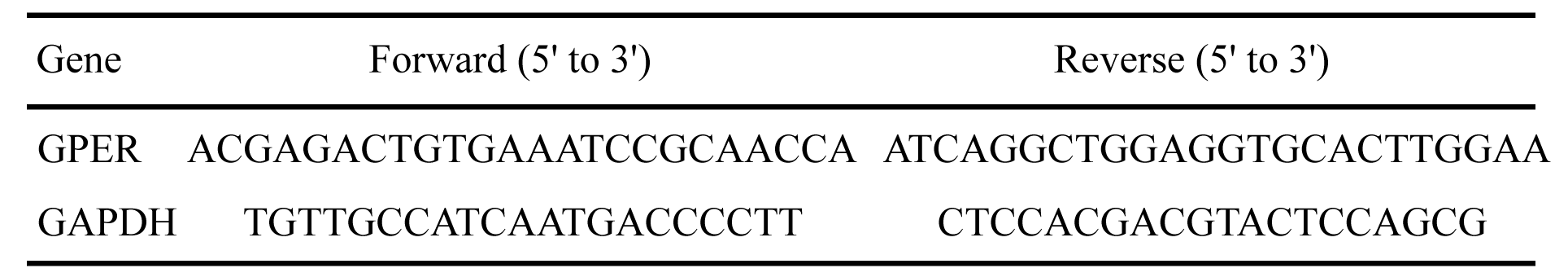

Supplement: Supplementary file 5 [file Image_4.tif]
